# Spermatogenesis Abnormalities following Hormonal Therapy in Transwomen

**DOI:** 10.1155/2018/7919481

**Published:** 2018-04-02

**Authors:** Sirachai Jindarak, Kasama Nilprapha, Taywin Atikankul, Apichai Angspatt, Pornthep Pungrasmi, Seree Iamphongsai, Pasu Promniyom, Poonpissamai Suwajo, Gennaro Selvaggi, Preecha Tiewtranon

**Affiliations:** ^1^Division of Plastic and Reconstructive Surgery, Department of Surgery, Faculty of Medicine, Chulalongkorn University, Bangkok, Thailand; ^2^Division of Pathology, Faculty of Medicine, Chulalongkorn University, Bangkok, Thailand; ^3^Department of Plastic Surgery, Institute of Clinical Sciences, Sahlgrenska Academy, University of Gothenburg, Sahlgrenska University Hospital, Gothenburg, Sweden; ^4^Preecha Aesthetic Institute, Bangkok, Thailand

## Abstract

**Objective:**

To measure spermatogenesis abnormalities in transwomen at the time of sex reassignment surgery (SRS) and to analyze the association between hormonal therapy duration and infertility severity.

**Design:**

Retrospective study.

**Setting:**

University hospital.

**Patients:**

One-hundred seventy-three transwomen who underwent SRS from January 2000 to December 2015.

**Interventions:**

All orchidectomy specimens were retrospectively reviewed and classified. History of hormonal therapy duration was retrieved from medical records.

**Main Outcome Measures:**

Histological examinations of orchidectomy specimens were performed to assess spermatogenesis.

**Results:**

One-hundred seventy-three orchidectomy specimens were evaluated. Histological examinations showed maturation arrest in 36.4%, hypospermatogenesis in 26%, Sertoli cell-only syndrome in 20.2%, normal spermatogenesis in 11%, and seminiferous tubule hyalinization in 6.4% of the specimens. Spermatogenesis abnormality severity was not associated with the total therapy duration (*P* = 0.81) or patient age at the time of surgery (*P* = 0.88). Testicular volumes and sizes were associated with spermatogenesis abnormality severity (*P* = 0.001 and *P* = 0.026, right testicle and left testicle, resp.).

**Conclusion(s):**

Feminizing hormonal treatment leads to reductions in testicular germ cell levels. All transwomen should be warned about this consequence, and gamete preservation should be offered before starting hormonal treatment.

## 1. Introduction

The therapeutic approach to gender dysphoria consists of many treatment options, including psychological support and hormonal and surgical treatments [1]. The goal of hormonal therapy is to suppress endogenous hormone levels and to replace exogenous sex hormone levels with those of the desired gender. Using hormonal therapy may lead to adverse effects such as venous thromboembolism, hypertriglyceridemia, weight gain, and gallstones [2]. Therefore, hormonal therapy should be administered under the care of qualified health professionals such as endocrinologists. For the best possible care, information regarding irreversible physical changes should be provided before the initiation of treatment [1]. Studies have shown that almost all Thai transsexuals self-medicate with hormonal therapy that includes hormones that are usually bought directly from the pharmacy without any prescription; therefore, transwomen are not warned about the loss of reproductive function [3, 4].

The objectives of this study were to identify spermatogenesis abnormalities in transwomen at the time of sex reassignment surgery (SRS) and to analyze the association between the level of infertility and duration of hormonal therapy.

## 2. Materials and Methods

This study was approved by the Committee of Research Affairs, Faculty of Medicine, Chulalongkorn University (project number 081/59). All orchidectomy specimens obtained during SRS at King Chulalongkorn Memorial Hospital from January 2000 until December 2015 were retrospectively reviewed by an experienced pathologist. Clinical data such as age at the time of surgery, duration of hormonal therapy, and hormonal therapy type were retrieved from the medical records. Our practice requires all patients to discontinue hormonal treatment for 4 weeks prior to surgery.

**Figure 1:**
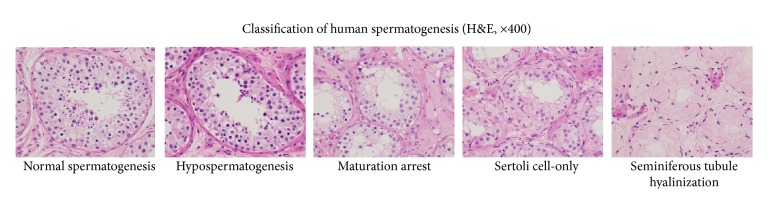
Classification of human spermatogenesis.

Orchidectomy specimens were examined histologically under light microscopy. Slides were evaluated for seminiferous tubules, germ cells, Sertoli cells, basement membrane thickness, and other abnormalities. Then, specimens were classified histologically as follows (Figure 1) [8]:

- *Normal testicular biopsy*: it is complete spermatogenesis during the entire biopsy and presence of normal intertubular tissue.
- *Hypospermatogenesis*: all stages of spermatogenesis are present but reduced to a varying degree. This includes a mixed pattern with some tubules showing Sertoli cells only or hyaline sclerosis, with other tubules containing complete spermatogenesis.
- *Maturation arrest*: it is complete arrest at a particular stage. This occurs most often at the spermatogonial or primary spermatocyte stage. If rare spermatids are present focally, then the lesion is classified as severe hypospermatogenesis rather than arrest.
- *Sertoli cell-only*: tubules contain only Sertoli cells and there is a complete absence of germ cells.
- *Seminiferous tubule hyalinization*: it is thickening of the peritubular membranes due to fibrosis and basement membrane-like material and the absence of intratubular germ cells and Sertoli cells.

In some cases, right and left testicular biopsy results showed discordant patterns. Regarding the clinical implications for fertility, patients who had discordant patterns in their testicular biopsy results were classified according to the less abnormal category. The number and percentage of each spermatogenesis classification were calculated using IBM SPSS Statistics 22. Categorical variables were compared using *χ*^2^ tests. One-way analysis of variance was used to compare continuous variables among these five histological groups. Kaplan-Meier curves were used to analyze the association of the duration of hormonal therapy and histological findings.

## 3. Results

A total of 173 transwomen underwent SRS at King Chulalongkorn Memorial Hospital between January 2000 and December 2015. The mean patient age on the day of the surgery was 26.09 ± 5.37 years. The mean testicular volume was 10.48 ± 7.46 ml in the right testicle and 9.89 ± 7.62 ml in the left testicle. The details of histopathological classification from the specimens are presented in Table 1. The most common abnormality was maturation arrest (63 patients; 36.4%). Normal spermatogenesis was found in 19 patients (11%). Three patients had discordant patterns as follows: normal and Sertoli cell-only, Sertoli cell-only and maturation arrest, and hypospermatogenesis and maturation arrest.

**Figure 2:**
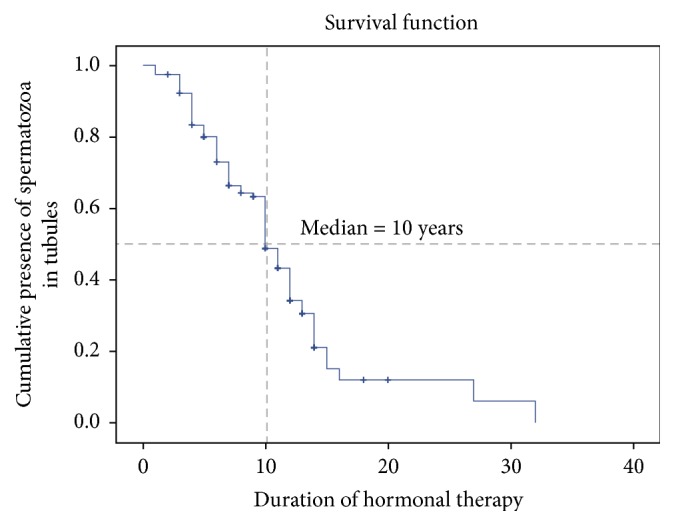
Kaplan-Meier curve shows the duration of hormonal therapy and the absence of spermatozoa in testicular tubules.

According to the available data, the mean duration of hormone use was 8.51 ± 4.67 years. The contraceptive pills used were Diane-35®, Sucee®, Yasmin®, Androcur®, Premarin®, and Progynova®; the contraceptive injection Progynon® was also used. Antiandrogens combined with estrogen were used for 18 patients (10.4%). Estrogen-only therapy was used for 38 patients (22%). Spironolactone and estrogen were used for one patient (0.6%). However, there were missing data regarding the types of hormones used for 57 patients (39%). The durations of hormonal treatments for each group are shown in Table 2. The mean ages between groups were not different (*P* = 0.88), and neither was the duration of hormonal exposure (*P* = 0.81). The Kaplan-Meier estimator in Figure 2 shows the duration of hormonal therapy that led to the absence of spermatozoa in testicular tubules (maturation arrest, Sertoli cell-only, and seminiferous tubule hyalinization). The mean duration was 10 years (95% confidence interval [CI], 9.03–10.97). The severity of abnormal spermatogenesis was directly associated with smaller volumes in both testes (*P* = 0.001 and *P* = 0.026).

## 4. Discussion

Although the number of scientific publications regarding the various treatments related to gender affirmation surgery is increasing, specific studies regarding spermatogenesis abnormalities following hormonal therapy in transwomen at the time of SRS are very limited, and the number of patients reported in these studies is very low [5, 6, 9, 10].

A case report of a transgender patient receiving estrogen showed a decreased amount of sperm in the semen after high-dose estrogen use for just 2 weeks [11]. Schneider et al. [6] examined the variations in spermatogenesis abnormalities in 46 testicular specimens from transwomen patients receiving the same antiandrogen and estrogen medications, as recommended by feminizing hormone guidelines, and their findings were similar to those of our study. However, in studies by Schneider et al. [6, 7], the proportion of those with normal (complete) spermatogenesis was higher than that of our study (26% versus 11%). This may be due to early hormonal exposure for Thai transwomen. When comparing the mean patient ages, we found that Thai transwomen involved in those studies who had undergone surgery were younger (26 versus 42 years). The estimated age of hormone initiation in our study was 17.59 ± 4.52 years; however, these data were lacking in the German study [7].

In the present study, morphological changes following prolonged hormonal usage were found in Leydig cells, Sertoli cells, and spermatogonia. The abnormality most commonly found was maturation arrest (36.4%), followed by hypospermatogenesis (26%) and Sertoli cell-only syndrome (20.2%). Seminiferous tubule hyalinization, which has the worst reproductive prognosis, was found in only 6.4% of patients. Eleven percent of patients had normal spermatogenesis. Our patients discontinued hormonal treatment before surgery, which may have affected spermatogenesis to some degree. Regardless, the results resembled those of the study by Schulze [9].  Table 3 compares our findings with those of similar studies. When comparing groups with spermatozoa still in the tubules (normal and hypospermatogenesis groups combined versus other groups: 37% versus 63%), the results were close to those of a study performed in Singapore (focal or normal spermatogenesis, 3 patients; absent spermatogenesis, 7 patients) [5].

Decreased testicular volume appeared to be related to the severity of the spermatogenesis abnormality (Table 2), and smaller-than-normal testes were observed in Thai males with abnormalities in the same age groups (right: 10.48 ml versus 17.2 ml; left: 9.89 ml versus 17.2 ml) [12]. This result was similar to that of the study by Schneider et al. [7] in which the testicular weight decreased with the severity of spermatogenesis and was correlated with serum testosterone level.

The 2009 guidelines for endocrine treatment of transsexual people stated variable timing of male sexual dysfunction after starting hormonal treatment [2]. Currently, the microdissection testicular sperm extraction technique (micro-TESE) can retrieve sperm at hypospermatogenesis maturation arrest and from the Sertoli cell-only group, but the success rate is low [13–17]. Therefore, we advocate informing transsexual patients before the commencement of hormonal therapy.

Because our study was retrospective, all patients were from Thailand, and patients were not followed by physicians during hormonal therapy. Data regarding hormonal usage were reported by patients; therefore, they might be somewhat inaccurate. To the best of our knowledge, this study is the largest series presenting spermatogenesis in a population of transwomen undergoing SRS following hormonal usage.

## 5. Conclusion

Feminizing hormonal treatment before SRS results in spermatogenesis abnormalities and loss of reproductive function. Maturation arrest was the most common abnormality encountered in our study (36.4%). Other abnormalities were hypospermatogenesis (26%) and Sertoli cell-only syndrome (20.2%). Normal spermatogenesis was present in only 11%. All transwomen should be advised about this adverse effect. Furthermore, cryopreservation of sperm before the initiation of hormonal treatment should be offered and discussed routinely.

## Figures and Tables

**Table 1:** Spermatogenesis classification of testicular specimens.

Classification	*n*	%
Normal spermatogenesis	19	11
Hypospermatogenesis	45	26
Maturation arrest	63	36.4
Sertoli cell-only syndrome	35	20.2
Seminiferous tubule hyalinization	11	6.4

Total	173	100

**Table 2:** Different hormonal treatments sorted by spermatogenesis classification.

	Normal	Hypospermatogenesis	Maturation arrest	SCO	Seminiferous tubule hyalinization	*P*
	*n* = 19	*n* = 45	*n* = 63	*n* = 35	*n* = 11	
	(11%)	(26%)	(36.4%)	(20.2%)	(6.4%)	
*Age*	25.95 ± 3.98	26.04 ± 3.55	26.62 ± 6.62	25.49 ± 4.74	25.45 ± 7.76	0.88

*Hormonal duration*	9.40 ± 3.18	8.49 ± 3.99	8.09 ± 5.24	8.35 ± 3.88	10 ± 8.79	0.81

*Testicular volume (ml)*						
Right	15.02 ± 2.50	12.14 ± 9.04	10.18 ± 6.07	7.43 ± 3.76	7.22 ± 3.50	0.001
Left	13.98 ± 10.0	10.38 ± 5.93	10.17 ± 9.06	7.21 ± 4.35	7.76 ± 5.16	0.026

SCO, Sertoli cell-only.

**Table 3:** Comparison of spermatogenesis abnormalities in transsexual women in various studies.

Year	Studies	*N*	Country	Spermatogenesis				
				Normal	Hypospermatogenesis	Maturation arrest	SCO	Seminiferous tubule hyalinization
1987	Thiagaraj et al. [5]	10	Singapore	*⟵* 30% *⟶*		*⟵* 70% *⟶*		
2013	Schneider et al. [6]	36	German	26%	28%	33%	11%	2%
2015	Schneider et al. [7]	108	German	24.07%	24.07%	35.17%	14.81%	1.85%
2017	Current study	173	Thailand	11%	26%	36.4%	20.2%	6.4%

SCO, Sertoli cell-only.
